# Male recombination map of the autosomal genome in German Holstein

**DOI:** 10.1186/s12711-020-00593-z

**Published:** 2020-12-14

**Authors:** Saber Qanbari, Dörte Wittenburg

**Affiliations:** Leibniz Institute for Farm Animal Biology (FBN), Institute of Genetics and Biometry, Wilhelm-Stahl-Allee 2, 18196 Dummerstorf, Germany

## Abstract

**Background:**

Recombination is a process by which chromosomes are broken and recombine to generate new combinations of alleles, therefore playing a major role in shaping genome variation. Recombination frequencies ($\theta$) between markers are used to construct genetic maps, which have important implications in genomic studies. Here, we report a recombination map for 44,696 autosomal single nucleotide polymorphisms (SNPs) according to the coordinates of the most recent bovine reference assembly. The recombination frequencies were estimated across 876 half-sib families with a minimum number of 39 and maximum number of 4236 progeny, comprising over 367 K genotyped German Holstein animals.

**Results:**

Genome-wide, over 8.9 million paternal recombination events were identified by investigating adjacent markers. The recombination map spans 24.43 Morgan (M) for a chromosomal length of 2486 Mbp and an average of ~ 0.98 cM/Mbp, which concords with the available pedigree-based linkage maps. Furthermore, we identified 971 putative recombination hotspot intervals (defined as $\theta$ > 2.5 standard deviations greater than the mean). The hotspot regions were non-uniformly distributed as sharp and narrow peaks, corresponding to ~ 5.8% of the recombination that has taken place in only ~ 2.4% of the genome. We verified genetic map length by applying a likelihood-based approach for the estimation of recombination rate between all intra-chromosomal marker pairs. This resulted in a longer autosomal genetic length for male cattle (25.35 cM) and in the localization of 51 putatively misplaced SNPs in the genome assembly.

**Conclusions:**

Given the fact that this map is built on the coordinates of the ARS-UCD1.2 assembly, our results provide the most updated genetic map yet available for the cattle genome.

## Background

Recombination is a process by which chromosomes are broken and recombine to produce new combinations of alleles, so-called haplotypes. Haplotypes possess specific genetic features, and thus play a major role in shaping genome variation. Crossover events are not uniformly distributed and regional rates of crossovers vary considerably across individual genomes and populations mostly because of the combined effects of mutation, recombination, and demographic history [1, 2]. Recombination frequencies between markers are used to construct genetic maps, which have important implications in genomic studies. High-resolution genetic maps are key elements of a successful fine-mapping program. Moreover, genetic linkage maps are valuable resources for the improvement of chromosome-level assemblies of whole-genome sequences and for comparative genome analyses to name just a few applications.

Genetic maps are built based either on tracing parent–offspring transmission [3, 4], sperm typing [5], or exploiting polymorphism data on a population scale [6, 7]. Given the controlled mating scheme in commercial animals, the primary strategy for the analysis of recombination has been through pedigree to benefit from the fully recorded genealogies. Such an approach traces transmission of haplotypes between pairs of loci from parents to offspring and infer genetic distance based on proportion of recombinant haplotypes.

Cattle have a vital role in the global food system and, given their economic importance, this species was among the first livestock to own a genetic map of recombination, which was built based on microsatellite markers [8, 9]. With the advent of single nucleotide polymorphism (SNP) arrays, studying recombination in farm animal genomes was accelerated with the motivation to assess accurate haplotype phasing and imputation that are required for implementing the genomic selection strategy. Subsequent recombination maps were then constructed at a higher resolution based on genotyping arrays in several beef [10, 11] and dairy breeds [12].

Holstein is the world’s most significant cattle breed with a prominent role in producing dairy products. Two recombination studies on Holstein cattle using medium-density genotypes were recently reported. Sandor et al. [13] characterized male bovine meiotic recombinations using 10,192 bulls from the Netherlands and 3783 bulls from New Zealand with 19,487 SNPs in common between the two groups. Ma et al. [4] reported a cattle sex-specific recombination map in a large pedigree of Holstein in the United States.

The recent genetic maps of cattle are built based on the arrays of SNPs that are mapped to the genome assembly UMD3.1 [14]. The emerging advances in long-read sequencing technologies have enabled a better alignment of sequence reads in the ARS-UCD1.2 re-assembly and improved overall continuity by reducing both gaps and inversions by more than 250-fold [15, 16]. The improved marker coordinates in the new assembly facilitate reliable haplotype phasing and imputation, and thus provide appropriate estimation of population genetics parameters such as inter-marker linkage disequilibrium and recombination frequencies, and eventually contribute to the success of gene mapping or genomic prediction projects.

In this study, we take the advantage of 50 K genotypes from a large pedigree of German Holstein cattle to construct an up-to-date genetic map, locate hotspot regions of recombination, and identify candidate genes that contribute to recombination. The novelty of our findings is twofold: (1) given the fact that this study uses the coordinates of the ARS-UCD1.2 assembly, it presents the most updated genetic map yet available for the cattle genome; and (2) we evaluate estimates of recombination rate between intra-chromosomal SNP pairs to identify misplaced markers. Furthermore, we introduce an optimization approach to verify the genetic map length.

## Methods

### Half-sib families from a large pedigree

This study used a large pedigree that includes 367,056 German Holstein cattle, and a subset of the animals have been genotyped for the genomic selection program in Germany. Data were provided by the German Evaluation Center, VIT (www.vit.de). The pedigree involved 1053 half-sib families with sires born between 1979 and 2017.

### Genetic material, quality control and imputation

Genetic data involved bi-allelic genotypes of 45,613 autosomal SNPs, which are mapped to the coordinates of the most recent ARS-UCD1.2 assembly (available at https://bovinegenome.elsiklab.missouri.edu/downloads/ARS-UCD1.2).

We used the PLINK v1.9 program [17] to clean the data for Mendelian inconsistencies both on the marker and individual levels. Markers that had a Mendelian inheritance error for more than 5% of the individual genotypes were removed. In total, 44,696 SNPs with a minor allelic frequency (MAF) higher than 0.01 and an average inter-marker distance of 55 kb were retained for the subsequent analyses. At the individual level, the Mendelian inconsistency threshold was set to 0.1. Genotypes with a Mendelian inheritance error were set to ‘NA’ and were imputed in a subsequent step. For the imputation of missing genotypes, we used the Eagle v2.3 software [18], which exploits available pedigree information and is capable of handling very large cohorts of individuals. Program parameters were set to the default values and were run chromosome-wise overnight in a multi-thread module.

### Recombination rates and genetic map positions

Recombination frequencies were estimated across 876 half-sib families, with sires having a minimum number of 39 progeny (see Additional file 1: Figure S1). Exploiting the genetic similarity between paternal half-sibs, the male recombination rate between marker pairs was assessed for each chromosome by the following methods.

### Deterministic approach

The deterministic approach of Ferdosi et al. [19] enabled inference of sire haplotypes from progeny genotypes, thus sire genotypes are not needed. The locations of recombination events and the most likely haplotype phases of a sire were reconstructed by grouping consecutive markers depending on the occurrence of opposite homozygous genotypes among the progeny. We used the implementation of this approach in the R package “hsphase” [20] and counted the number of crossovers between adjacent markers in each half-sib family. The proportion of recombinant haplotypes in a marker interval was then averaged over all the families to estimate recombination rate. Given the close proximity of markers and assuming no interference between successive crossovers, estimated recombination rates were directly converted into genetic distances in Morgan (M) units. The hsphase method is limited to adjacent markers only. In addition, these estimates were considered for the evaluation of hotspot regions.

### Likelihood-based approach

Let $p$ denote the number of markers on a chromosome. The recombination rate $\theta_{i,j}$ between each pair of markers $i$ and $j$, ($i, j = 1, \ldots ,p; i < j$) was estimated using an expectation–maximization approach which relies on likelihood theory [21–23]. This approach uses sire haplotypes that have been reconstructed within each half-sib family by hsphase and progeny genotypes. Estimating recombination frequency across all intra-chromosomal marker pairs allowed the identification of markers that are misplaced in the current genome assembly. For this purpose, SNPs with a markedly high recombination rate with the neighboring markers were identified following Hampel et al. [23]. Briefly, the mean recombination rate of $\theta_{i, j + 1} , \ldots ,\theta_{i, j + 30}$ was calculated for all SNPs $i = 1, \ldots ,p - 30$, and the mean of $\theta_{i - 30,i} , \ldots ,\theta_{i - 1,i}$ was taken for $i = p - 29, \ldots ,p$. If the mean recombination rate exceeded the chromosome-wide 99% quantile, the SNP was considered as a misplaced candidate, which was confirmed through subsequent visualization of the increased recombination rate with the following SNPs on a heatmap.

In order to account for possible genotype errors, and to reduce the influence of statistical uncertainty on parameter estimates, we developed a smoothing approach to approximate genetic distances between adjacent markers. Instead of converting recombination rate between adjacent markers only, we considered all the estimates $\widehat{\theta }_{i,j} \le 0.05$ in a quadratic optimization approach. Then, only a linear relationship between recombination rate and genetic distance was assumed to hold. Let $d_{k}$ denote the genetic distance between markers $k$ and $k + 1$ in M units. As genetic distances are additive, e.g. $d_{1} + d_{2} + d_{3}$ is the genetic distance that corresponds to $\theta_{1,4} \le 0.05$, the optimization problem was specified in terms of squared deviations:

$$ {\text{min}}_{{d_{1} , \ldots ,d_{p - 1} }} \left\{ {\mathop \sum \limits_{{\begin{array}{*{20}c} {i,j = 1} \\ {i < j} \\ {\widehat{\theta }_{i,j \le 0.05} } \\ \end{array} }}^{p} \left( {\widehat{\theta }_{i,j} - \mathop \sum \limits_{k = i}^{j - 1} d_{k} } \right)^{2} } \right\} {\text{s}}.{\text{t}}.{ }d_{k} \ge 0,k = 1, \ldots ,p - 1. $$

The genetic length of a chromosome was derived as the sum over interval lengths. All steps of the likelihood-based approach were implemented in the R package “hsrecombi” version 0.3.1 that is available at CRAN [24].

### Genome-wide association study for recombination frequency

A linear mixed model for genome-wide association analysis (GWAS) implemented in the GCTA program [25] was used to identify loci that have large effects on recombination activity. GWAS was conducted on all sires for which the genotype was available. The phenotype for each sire was estimated by averaging the number of recombination events across progeny. We tested the association between each SNP and the phenotype “recombination frequency” using the following model equation:

$$ {\mathbf{y}} = {\mathbf{Xg}} + {\mathbf{Za}} + {\mathbf{e}}, $$

where ${\mathbf{y}}$ is the vector of phenotypes for 875 sires, ${\mathbf{X}}$ is the design matrix of fixed effects ${\mathbf{g}}$, including a population mean and the additive effect of the candidate SNP, ${\mathbf{Z}}$ is the design matrix for a random animal effect ${\mathbf{a}}$ with ${\mathbf{a}}\sim N\left( {0, {\mathbf{G}}\sigma_{a}^{2} } \right)$ with ${\mathbf{G}}$ the genomic relationship matrix of sires, and ${\mathbf{e}}$ is the vector of independent and identically distributed residuals.

## Results and discussion

### Mendelian inconsistency

**Fig. 1:**
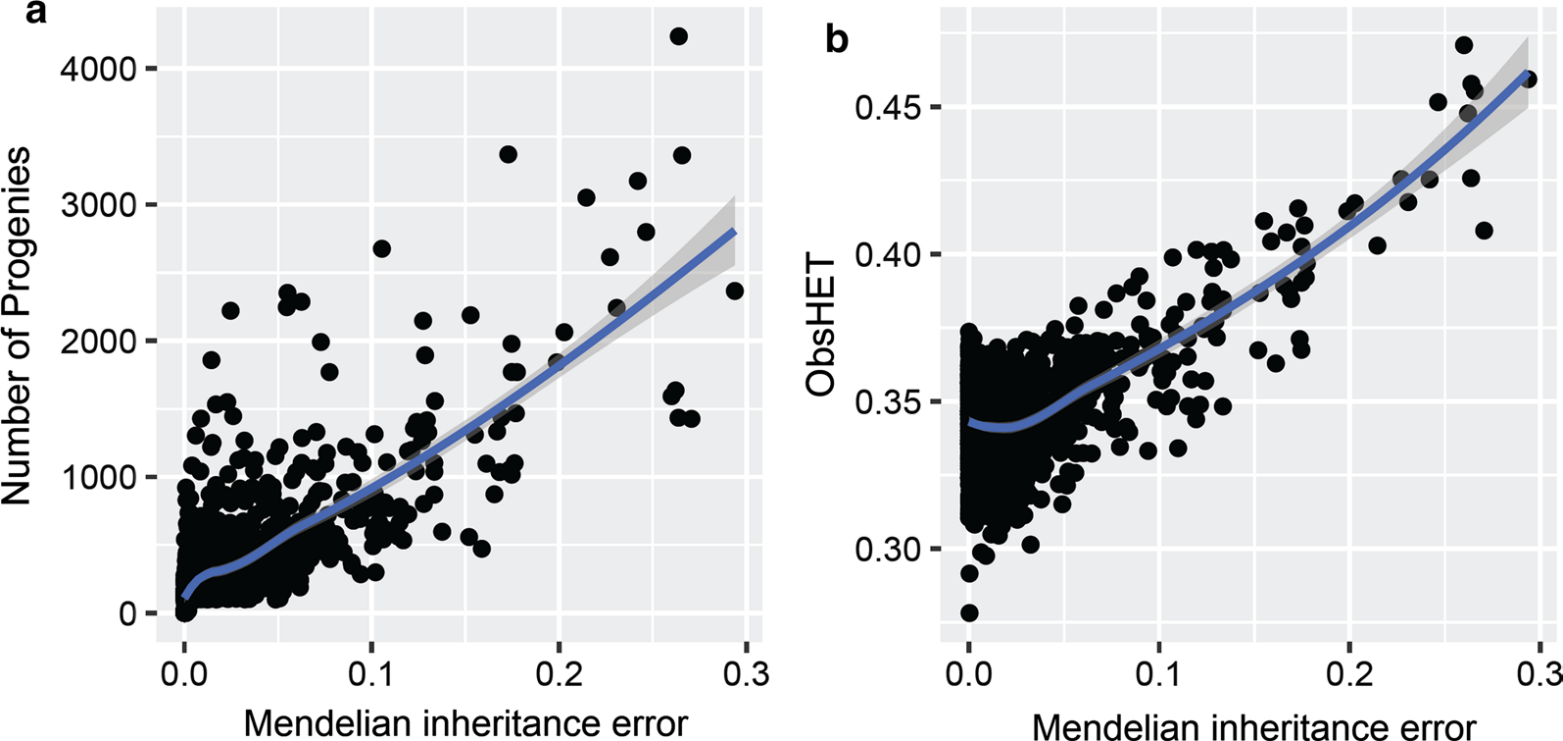
Mendelian inconsistency in the genotype data. **a** Distribution of Mendelian inheritance errors by number of progeny per sire. **b** Relationship between the observed heterozygosity and Mendelian inheritance error in sires

Checking genotype data for Mendelian inconsistency is a necessary step to estimate recombination frequencies. A Mendelian inheritance error is defined as the discrepancy between the genotype and pedigree data of two related animals (e.g., parents and offspring). This may result from an error in the recorded pedigree, from genotyping errors, or from mixing up DNA samples, and in very rare cases from mutations [26]. We conducted an exploratory analysis on Mendelian inconsistency in the marker dataset before investigating recombination. A subset of 69 sires showed a Mendelian inconsistency rate higher than 10% for the genotypes and these were excluded from subsequent analyses. As expected, we observed a positive association of the Mendelian inheritance error with the number of progeny genotyped per sire, which is obviously explained by the number of assessments performed per sire to verify genotypes between sire-offspring (Fig. 1a). Mendelian inconsistency was also positively correlated with sire heterozygosity (Fig. 1b).

### Construction of the male recombination map

We built the male recombination map based on genotypes for 44,696 autosomal SNPs with the coordinates derived from the most recent cattle genome assembly. To ensure accurate estimates of recombination frequencies, sires with more than 39 progenies were excluded. Recombination rates were estimated across 876 half-sib families with a maximum number of 4236 progenies (see Additional file 1: Figure S1).

#### Deterministic approach

By tracking paternal meiosis through sire/offspring genotypes, over 8.9 million recombination events were identified genome-wide in the pairwise comparison of adjacent markers. The recent genetic map in US Holstein cattle was constructed across 8.5 million paternal and maternal recombination events; on average, i.e. 36 recombination events per individual across the genome [4].

**Fig. 2:**
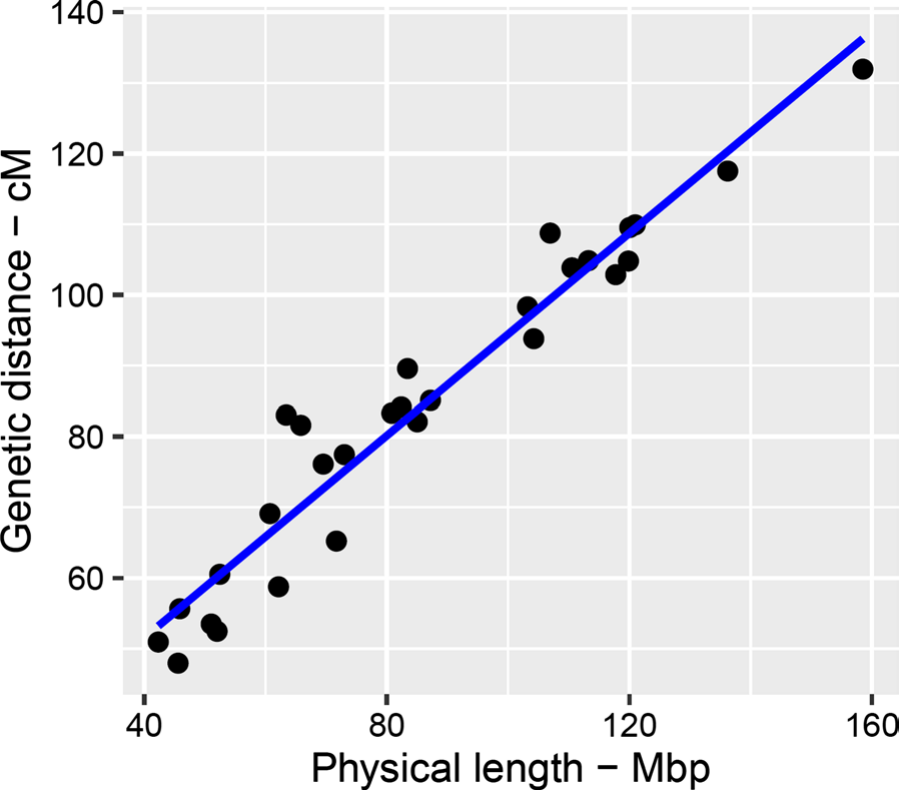
Genetic versus physical length of the bovine autosomes. The Pearson correlation r = 0.967 was estimated between the physical and genetic lengths

The recombination map spans 24.43 M on the autosomal genome (Fig. 2). Based on the bovine ARS-UCD1.2 assembly, the total physical length of the autosomes was 2.486 Gbp. The average recombination distance was approximately ~ 0.98 cM per million bp (cM/Mbp). This is fairly consistent with the most recent linkage maps built by Ma et al. [4] and Sander et al. [13] who reported autosomal genome lengths of 25.5 and 25.7 M, respectively, for male cattle. In cattle, the male recombination map has been reported to be 10% longer than the female map [12]. As a general trend genome-wide, we observed significantly higher recombination rates on short chromosomes than on long chromosomes (P-value = 0.0003 two-sample t-test). Accordingly, we found the longest genetic map for *Bos taurus* chromosome (BTA19), which spanned on average ~ 1.31 cM/Mbp, versus ~ 0.83 cM/Mbp for BTA1. The full list of recombination frequencies between pairs of adjacent markers is in Additional file 2: Table S1 and local recombination rates chromosome-wise are in Additional file 3: Figure S2. The emerging picture is that the recombination activity increased across the middle part of most chromosomes and dropped towards both chromosome ends given the acrocentric nature of bovine autosomes and the underlying differences between the structure of centromeric and telomeric DNA.

#### Likelihood-based approach

The likelihood-based approach generated estimates of $\theta$ for all intra-chromosomal marker pairs if at least one sire was double heterozygous. For instance, 2902 out of the 2911 SNPs considered on BTA1 yielded only 4,116,903 estimates of recombination rate. In general, the number of estimates was smaller than expected from the SNP number (e.g., 4,209,351 on BTA1) since double heterozygosity was observed only for 98% of all eligible SNP pairs on each chromosome. Note that sires with long runs of homozygosity can still be effective in the estimation of local recombination rates if heterozygous loci occasionally appear. Estimates of $\theta$ were based on the genotypes of at least 39 or at most 212,823 progeny across families. For instance, on average 42,422 progeny were involved for BTA1. The total genetic map length estimated by using the likelihood-based approach was 25.35 cM, which is in perfect agreement with the most recent linkage maps built by Ma et al. [4] and Sander et al. [13]. On average, the genetic length was 1.05 times longer with the likelihood-based approach than with the deterministic approach (see Table 1) in which only a fraction of the available information was exploited (i.e., 2910 estimates of genetic distances between SNPs on BTA1). The relationship between genetic and physical positions is shown chromosome-wise in Additional file 4: Figure S3. Whereas a linear relationship was obtained for some chromosomes (e.g., BTA25 and 27), an S-shaped curve was found for most of the other chromosomes, which is explained by the variation of local recombination rates as stated above. The visible gaps on BTA10, 27 and 29 are in Table 1.

**Table 1:** A summary of the statistics of the genetic map for bovine autosomes

Chr	nSNP	bp	Gap (bp)	Space (kb)	nRec	D (M))	cMMb^−1^ (D)	L (M)	cMMb^−1^ (L)
1	2911	158,517,589	497,531	54.16	483,569	1.319	0.832	1.269	0.801
2	2355	136,218,516	669,500	57.82	430,718	1.175	0.863	1.156	0.848
3	2190	120,957,517	799,833	55.15	402,820	1.099	0.909	1.152	0.952
4	2164	119,841,669	467,518	55.32	384,044	1.048	0.874	1.088	0.907
5	1868	120,055,511	727,739	64.24	401,460	1.095	0.912	1.142	0.951
6	2213	117,744,633	554,476	53.14	376,980	1.029	0.874	1.046	0.889
7	1945	110,528,375	870,522	56.66	380,527	1.038	0.939	1.062	0.961
8	2104	113,252,524	498,996	53.82	384,270	1.048	0.926	1.030	0.909
9	1761	104,228,150	663,781	59.18	343,786	0.938	0.900	0.973	0.934
10	1852	103,192,471	2,750,827	55.70	360,313	0.983	0.953	1.036	1.004
11	1937	106,932,443	700,224	55.17	398,538	1.087	1.017	1.067	0.998
12	1484	87,186,356	1,266,681	58.64	311,932	0.851	0.976	0.887	1.017
13	1522	83,402,661	726,077	54.53	328,307	0.896	1.074	0.940	1.127
14	1544	82,366,657	575,373	53.20	308,463	0.842	1.022	0.883	1.072
15	1498	85,007,180	727,620	56.38	300,883	0.821	0.966	0.862	1.014
16	1437	80,814,937	693,107	56.11	305,304	0.833	1.031	0.881	1.091
17	1390	72,986,398	779,268	52.45	282,258	0.775	1.061	0.802	1.099
18	1173	65,793,776	872,112	55.58	299,098	0.816	1.240	0.822	1.249
19	1189	63,394,562	674,383	53.01	304,320	0.830	1.310	0.914	1.441
20	1385	71,677,629	546,922	51.56	239,093	0.652	0.910	0.679	0.948
21	1192	69,498,436	737,425	57.95	278,990	0.761	1.095	0.779	1.121
22	1100	60,710,593	465,820	55.08	253,327	0.691	1.138	0.736	1.212
23	943	52,433,171	625,714	55.50	221,849	0.605	1.154	0.637	1.214
24	1091	62,127,707	427,626	56.72	215,397	0.588	0.946	0.654	1.052
25	865	42,292,572	234,159	48.89	186,752	0.510	1.205	0.566	1.338
26	944	51,990,348	367,274	54.36	192,294	0.525	1.009	0.578	1.113
27	862	45,553,866	1,148,867	52.75	175,767	0.480	1.053	0.553	1.214
28	840	45,834,413	338,885	54.34	203,987	0.557	1.214	0.553	1.206
29	937	51,028,789	1,374,496	54.06	196,068	0.535	1.048	0.607	1.190
#	44,696	2,485,569,449	2,750,827	55.46	8,951,114	24.426	0.983	25.354	1.020

bp: chromosome length in base pairs; Gap: maximum gap size between pairs of adjacent markers; Space: inter-marker space; nRec: number of cross-overs detected; D (M): genetic length in Morgan estimated based on deterministic approach; L (M): genetic length in Morgan estimated with the likelihood-based approach. #Depending on the parameter, either mean (Space, cMMb^−1^), maximum (Gap) or sum (nSNP, BP, nRec, Morgan) is represented

### Candidates of misplaced SNPs

Although we used coordinates of the most recent genome assembly, we draw attention to the fact that some remaining errors in the genome assembly such as misplaced markers may still lead to erroneous assessment of recombination frequencies and a spurious hotspots landscape [27]. This suggests that the identified hotspots could be targets for further investigations to correct the genome assembly. We followed two strategies in parallel to position misplaced markers and to circumvent the false-positive recombination assessments.

**Fig. 3:**
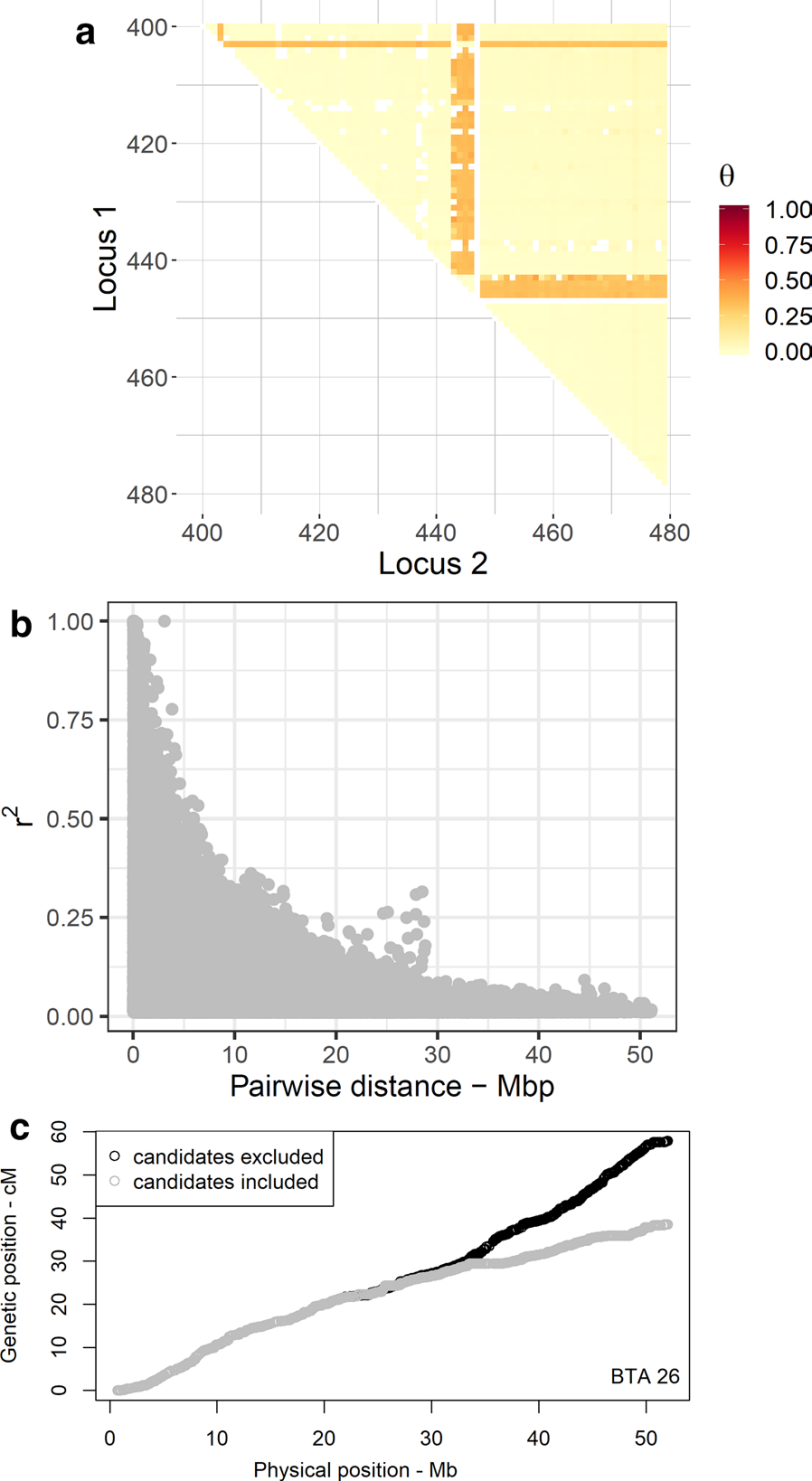
Localization of putatively misplaced candidates on BTA26. **a** Heatmap of recombination rate with misplaced candidates. The selected window of 80 SNPs ranges from 23.08 to 27.53 Mbp. A single SNP (on the top) and a cluster of four SNPs (in the middle) are misplaced in the underlying genome assembly. Missing values are filled with white color. **b** Visualizes the inflated LD between physically distant loci. A cluster of inflated LD appears between markers mapped as far as ~ 25 Mbp from each other. Two SNPs ARS-BFGL-NGS-119202 and ARS-BFGL-NGS-58121 were mainly involved in shaping the outlaying pattern of LD. **c** The genetic distances of SNPs approximated with the likelihood-based approach before (grey) and after removing misplaced candidates (black) by physical distance

We searched for markers with markedly high recombination rates to the neighboring markers according to Hampel et al. [23]. In total, we found that 51 of the SNPs mapped on 18 chromosomes were putatively misplaced in the ARS-UCD1.2 assembly. As an example, on BTA26, single SNPs that mapped at positions 23.16 and 51.58 Mbp together with a cluster of four SNPs at 25.65–25.76 Mbp revealed increased recombination rates with all other SNPs (see Fig. 3a). The full list of misplaced candidates is in Additional file 5: Table S2.

Alternatively, we used the linkage disequilibrium (LD) between markers to verify putatively misplaced SNPs. To this end, sire haplotypes were reconstructed in hsphase, and the LD that was estimated as the allelic correlation (r^2^) between pairs of markers was plotted as a function of physical distance. The pattern of LD decay revealed clusters of inflated LD between loci that were physically mapped as far as several millions of bp from each other, which indicates misplaced markers even in the recent assembly (Fig. 3b). LD analysis successfully detected the misplaced candidates that were detected by the likelihood-based approach. The subsequent removal of misplaced SNPs resulted in a smooth decay of LD as a function of inter-marker distance, which provided evidence that the methodology used to detect these markers was appropriate.

Excluding SNPs with a putatively wrong physical position is also essential for a proper approximation of the genetic distances. For example, the genetic lengths of BTA26 and 23 were estimated to be respectively 49% and 2% longer when misplaced candidates were excluded (e.g., see Fig. 3c). In contrast, the estimated genetic lengths of BTA1 and 28 declined by 5% and 2%, respectively, after removing the misplaced candidates. The genetic length of the remaining chromosomes was almost unaffected. Thus, we argue that the application of the likelihood-based approach followed by a verification step based on LD analysis can be efficiently used to screen marker panels of different densities for putatively misplaced SNPs. Improved map coordinates will eventually contribute to the success of gene mapping studies that are conducted based on available genotyping arrays in different species.

### Deterministic versus likelihood-based approach

We applied two approaches to estimate genetic map length and found that the lengths obtained differed by about 4% (almost 1 M). A simulation study on the verification of the two approaches is provided in Additional file 6. The accuracy of the genetic distances that were obtained from the likelihood-based approach was higher than that of the deterministic estimates with a difference in total genetic length of the same order of magnitude as in the real data analysis. However, both approaches underestimated the simulated genetic distances, and more research is needed to improve these estimation methods. Still, we decided to present both approaches since they possess different advantages: the deterministic approach allowed the elucidation of hotspot intervals and enabled identification of genome regions associated with recombination activity. Although, in principle, the likelihood-based approach is also applicable for the verification of hotspot regions, only this approach made it possible to clearly pinpoint putatively misplaced SNPs in the genome assembly.

### Landscape of recombination hotspots

**Fig. 4:**
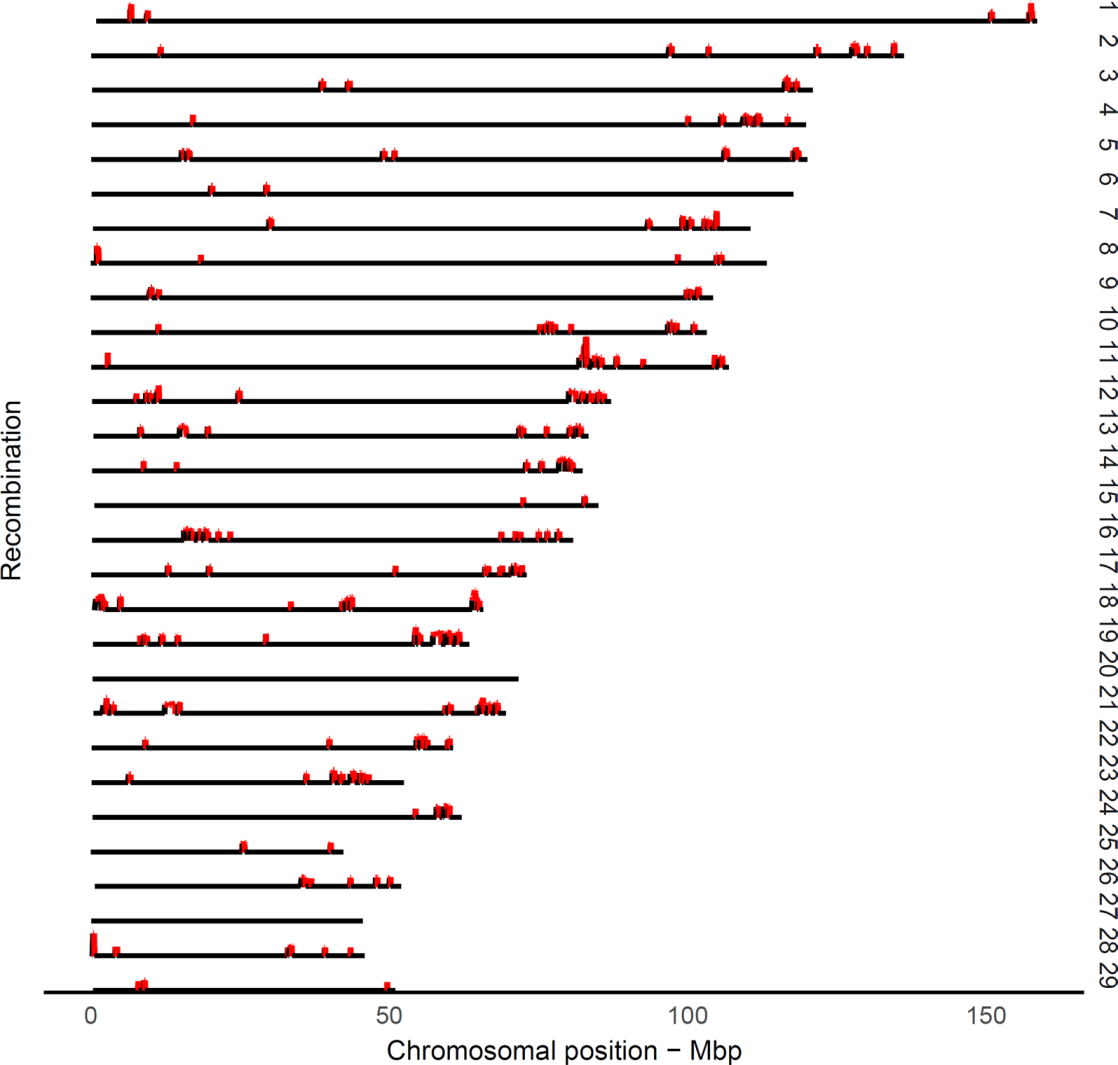
Genome-wide landscape of recombination hotspot intervals. The putative hotspot interval was defined as having a recombination rate with more than 2.5 standard deviations greater than the genome-wide mean of recombination rates

Following Ma et al. [4], we defined a hotspot region as a region with a recombination rate exceeding 2.5 standard deviations from the genome-wide average of recombination rates. The landscape of highly recombinant intervals or hotspot regions emerged as sharp and narrow peaks that occurred for a small proportion of the genome (Fig. 4). As expected, hotspot regions were non-uniformly distributed across the genome, which is consistent with previous observations in other mammals [2, 7]. After removing spurious hotspot intervals due to misplaced SNPs, a panel of 971 putative hotspot intervals were identified that represented ~ 5.8% of the recombination that occurred in only ~ 2.4% of the genome (see Additional file 1: Table S1). Previous studies in cattle based on medium-density SNP panels reported rather similar numbers of putative hotspot intervals. For example, Ma et al. [4] detected 1792 male putative hotspot regions that represented 3% of the genome. Another study identified 1378, 1295, and 1317 hotspot regions in Jersey, Brown Swiss, and Ayrshire breeds, respectively [12]. In contrast, studies on recombination in the human and mouse genomes that used full re-sequencing or very dense genotyping data, identified ~ 33,000 [7] and ~ 47,000 [2] hotspots, respectively, given that the genome size in mammals is comparable. In humans, ∼80% of the crossovers map to ∼10 to 20% of the genome, where the typical length of hotspots is less than 5 kb [28]. It is worth noting that, in our study, hotspots were localized by using a medium-density panel of SNPs with an average inter-marker space of 55 kb, thus they cannot be directly compared to the hotspots detected by using sequence data in the human or mouse genome. Therefore, we used the term “hotspot interval” instead of “hotspot” to report highly recombinant regions. In addition, it should be noted that the variation between physical inter-marker intervals (see Table 1) has not been taken into account in the definition of hotspot intervals, where the larger gaps between adjacent markers are expected to result in increased recombination frequencies.

### Genomic regions associated with recombination frequency

We conducted a GWAS including 875 sires of half-sib families to detect genes that influence the trait “recombination frequency”. The genome-wide number of crossovers that occur between adjacent SNP pairs of each sire was treated as the phenotype. Given the fact that all sires were genotyped on the same panel of markers, we ruled out the possible effect of SNP density on the number of crossovers counted for each sire. Over 8.9 million recombination events were detected across the half-sib families which was a sufficiently large number to result in accurate estimates of recombination frequencies.

**Fig. 5:**
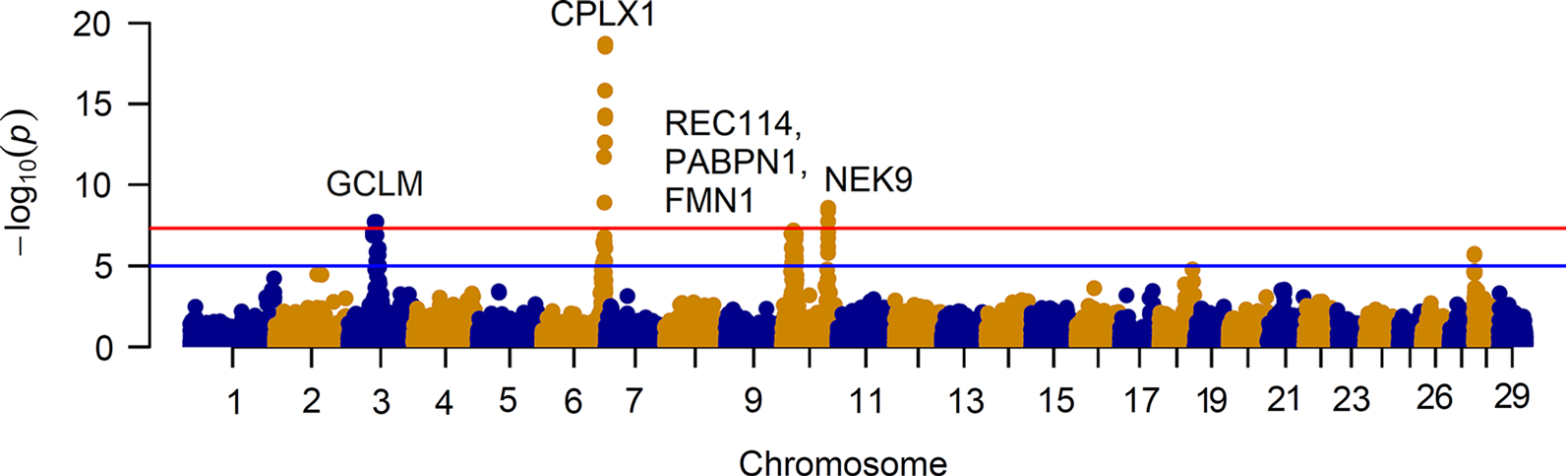
A schematic representation of the underlying genetics that controls male recombination rates in the genome of Holstein cattle. The genome-wide significance level of 2.1 × 10^−7^ is indicated by the horizontal red line. Marker positions derived from ARS-UCD1.2 assembly were used for plotting

Our results show that there are standing alone signals on a number of chromosomes and that the strongest candidates are on BTA3, 6 and 10 (Fig. 5), which is consistent with previous reports in cattle [4, 12, 13]. Applying a Bonferroni correction, a genome-wide threshold of 2.1 × 10^− 7^ was set to identify significant signals. In total, 24 significant SNPs (corrected P-value ≤ 0.01), emerged with a strong effect on recombination activity (Table 2). The signal with the strongest effect (P = 1.89 × 10^−19^) corresponded to SNP ARS-BFGL-NGS-110507 on BTA6, which co-localized with a region that contains three candidate genes, *CPLX1*, *GAK* and *PCGF3*. *CPLX1* encodes a protein that belongs to a family of cytosolic proteins, which have a role in synaptic vesicle exocytosis and are reported to be associated with sex-related variation of recombination frequency in sheep [29] and cattle [4, 12]. We also mapped two strong candidate SNPs on BTA10. The first signal had a maximum peak at SNP Hapmap47676-BTA-61231 (P = 6.75 × 10^−8^) and was localized in the vicinity of several meiosis-related genes including *REC8*, *REC114*, and *FMN1*. REC8 is a key component of the meiotic cohesion complex, and is associated with recombination activity in cattle [4, 12, 13], mouse [30] and Red Deer [31]. The second significant signal was associated with SNP BTA-78285-no-rs (P = 2.68 × 10^−9^ on BTA10 and overlapped with the *NEK9* gene. NEK9 mediates cell cycle progression that is essential for interphase progression during oocyte formation [32, 33] and is associated with crossover interference levels [34] and recombination activity in mammals [4]. Another significant signal peaked at SNP Hapmap59096-rs29024776 (P = 1.97 × 10^−8^) in a gene-rich region on BTA3. Although a statistical association with a QTL has already been reported [4], a biological association with the neighboring candidate genes needs to be established.

**Table 2:** Summary of the statistics of SNPs associated with recombination frequency

Chr	SNP	bp	Frequency	P-value	BF	Candidate gene
3	INRA-598	45,930,136	0.21	8.67 × 10^−8^	0.003	
3	ARS-BFGL-NGS-112152	45,978,363	0.21	1.42 × 10^−7^	0.006	
3	Hapmap59096-rs29024776	49,181,271	0.21	1.97 × 10^−8^	0.001	*GCLM*
3	Hapmap58808-rs29017431	52,452,892	0.20	1.98 × 10^−8^	0.001	
3	INRA-170	52,783,346	0.20	1.32 × 10^−7^	0.006	
6	ARS-BFGL-NGS-28350	114,972,434	0.26	1.88 × 10^−12^	0.000	
6	ARS-BFGL-NGS-18656	115,871,184	0.39	1.28 × 10^−9^	0.000	
6	ARS-BFGL-NGS-104112	115,942,196	0.30	1.73 × 10^−7^	0.007	
6	ARS-BFGL-NGS-61359	116,788,648	0.28	2.36 × 10^−13^	0.000	
6	ARS-BFGL-NGS-10037	117,015,280	0.27	1.50 × 10^−16^	0.000	
6	ARS-BFGL-NGS-112242	117,124,190	0.29	7.68 × 10^−15^	0.000	
6	BTB-00284077	117,271,685	0.27	5.18 × 10^−15^	0.000	
6	ARS-BFGL-NGS-117763	117,368,760	0.29	2.89 × 10^−19^	0.000	
6	ARS-BFGL-NGS-110507	117,390,034	0.29	1.89 × 10^−19^	0.000	*CPLX1, GAK, PCGF3*
10	ARS-BFGL-NGS-99693	17,886,463	0.61	1.09 × 10^−7^	0.004	
10	Hapmap47676-BTA-61231	21,768,228	0.15	6.75 × 10^−8^	0.003	
10	ARS-BFGL-NGS-19822	22,284,939	0.42	2.09 × 10^−7^	0.009	
10	ARS-BFGL-NGS-42815	25,998,000	0.59	1.08 × 10^−7^	0.004	*REC114*
10	ARS-BFGL-NGS-118433	26,023,168	0.59	1.08 × 10^−7^	0.004	
10	BTB-00438757	86,199,353	0.40	1.85 × 10^−8^	0.001	
10	Hapmap57084-ss46526565	86,260,186	0.40	4.17 × 10^−9^	0.000	
10	BTB-00438922	86,284,751	0.40	2.05 × 10^−7^	0.009	
10	BTA-78285-no-rs	86,322,591	0.55	2.68 × 10^−9^	0.000	*NEK9*
10	UA-IFASA-7857	86,379,951	0.56	8.37 × 10^−8^	0.003	

BF: Bonferroni adjusted P-value for multiplicity

The above-mentioned regions are implicated in recombination variation at the individual level in humans, cattle and mice, which suggests a common genetic architecture of recombination activity in mammals. The variation observed in genome-wide recombination frequency among sires can be used as an opportunity to maintain the genomic diversity of intensively selected dairy cattle, which has been shrinking for decades.

## Conclusions

We present a bovine genetic map with a medium SNP density resolution based on a large pedigree of German Holstein animals. The deterministic approach used recombination frequencies between adjacent markers to construct the genetic map that spans 24.4 M with an average length of ~ 0.98 cM/Mbp-1. We identified 971 highly recombinant marker intervals/hotspot regions that were non-uniformly distributed across ~ 2.4% of the genome. The likelihood-based approach resulted in a genetic length of 25.3 M, which fits better with the available linkage map lengths. Taking benefit of all pairwise recombination estimates, the likelihood-based approach was able to localize 51 SNPs that were putatively wrongly assigned on the physical map. The genome-wide association study identified several candidate loci including *REC8*, *REC114*, *FMN1* and *CPLX1* that affect recombination frequency. Our results successfully validated those of previous reports on the genetics that underlies recombination activity in cattle. Given the fact that this map is built on the coordinates of the ARS-UCD1.2 assembly, our results provide the most updated genetic map yet available for the cattle genome. The map presented in this study will be useful for both breeders and researchers and will support further investigation of the genome of this economically important species. The R package and workflow provided will allow to estimate the length of the genetic map of other breeds and thus will facilitate future comparisons of the genome characteristics between breeds.

## Supplementary Information

**Additional file 1: Figure S1. **Number of progeny per sire of half-sib families.

**Additional file 2: Table S1.** Genetic-map coordinates. The table contains the genetic-map coordinates that were estimated from deterministic (cM_deterministic) and likelihood-based (cM_likelihood) approaches in Holstein cattle. Marker physical coordinates (Mbp_position) are based on the ARS-UCD1.2 genome assembly. Furthermore, recrate_adjacent_deterministic denotes the recombination rate between adjacent markers based on the deterministic approach.

**Additional file 3: Figure S2. **Illustration of the recombination rate along the chromosomes. The figure shows the relationship between recombination rate between adjacent markers based on the deterministic approach and the relative physical position for each chromosome.

**Additional file 4: Figure S3. **Physical genetic maps for each chromosome. The figure shows the relationship between physical and genetic-map coordinates for each chromosome.

**Additional file 5: Table S2. **Panel of misplaced candidates in the ARS-UCD1.2 genome assembly**. **The table lists all the markers that are putatively misplaced in the underlying genome assembly, as revealed by the likelihood-based approach. Physical position (bp) corresponds to ARS-UCD1.2 genome assembly; the index refers to consecutive numbering of SNPs to facilitate the identification of clusters.

**Additional file 6. **Description of the simulation study to compare genetic map positions derived from the deterministic and likelihood-based approach [20–24, 35, 36].

## Data Availability

The data supporting the findings of this study was provided by the German Evaluation Center (VIT, Verden) and their access is restricted by the provider to be publicly available. Permission for data access was granted by the Association for Bioeconomy Research (FBF, Bonn). The R package hsrecombi version 0.3.1 is available at CRAN; it provides tools for estimating recombination rates between marker pairs, identifying candidates of misplaced markers and approximating genetic-map positions.
