# Correction to: Male recombination map of the autosomal genome in German Holstein

**DOI:** 10.1186/s12711-021-00604-7

**Published:** 2021-01-21

**Authors:** Saber Qanbari, Dörte Wittenburg

**Affiliations:** Leibniz Institute for Farm Animal Biology (FBN), Institute of Genetics and Biometry, Wilhelm-Stahl-Allee 2, 18196 Dummerstorf, Germany

## Correction to: Genet Sel Evol (2020) 52, 73 10.1186/s12711-020-00593-z

After publication of the original article [1], the authors reported an error in the contribution declaration. This is to notify that both authors have contributed equally and co-correspond this article.
