# Supplementary figures and images for: Male recombination map of the autosomal genome in German Holstein
Source: Genet Sel Evol. 2020 Dec 14;52:73. doi: 10.1186/s12711-020-00593-z (PMC7734841; doi:10.1186/s12711-020-00593-z)

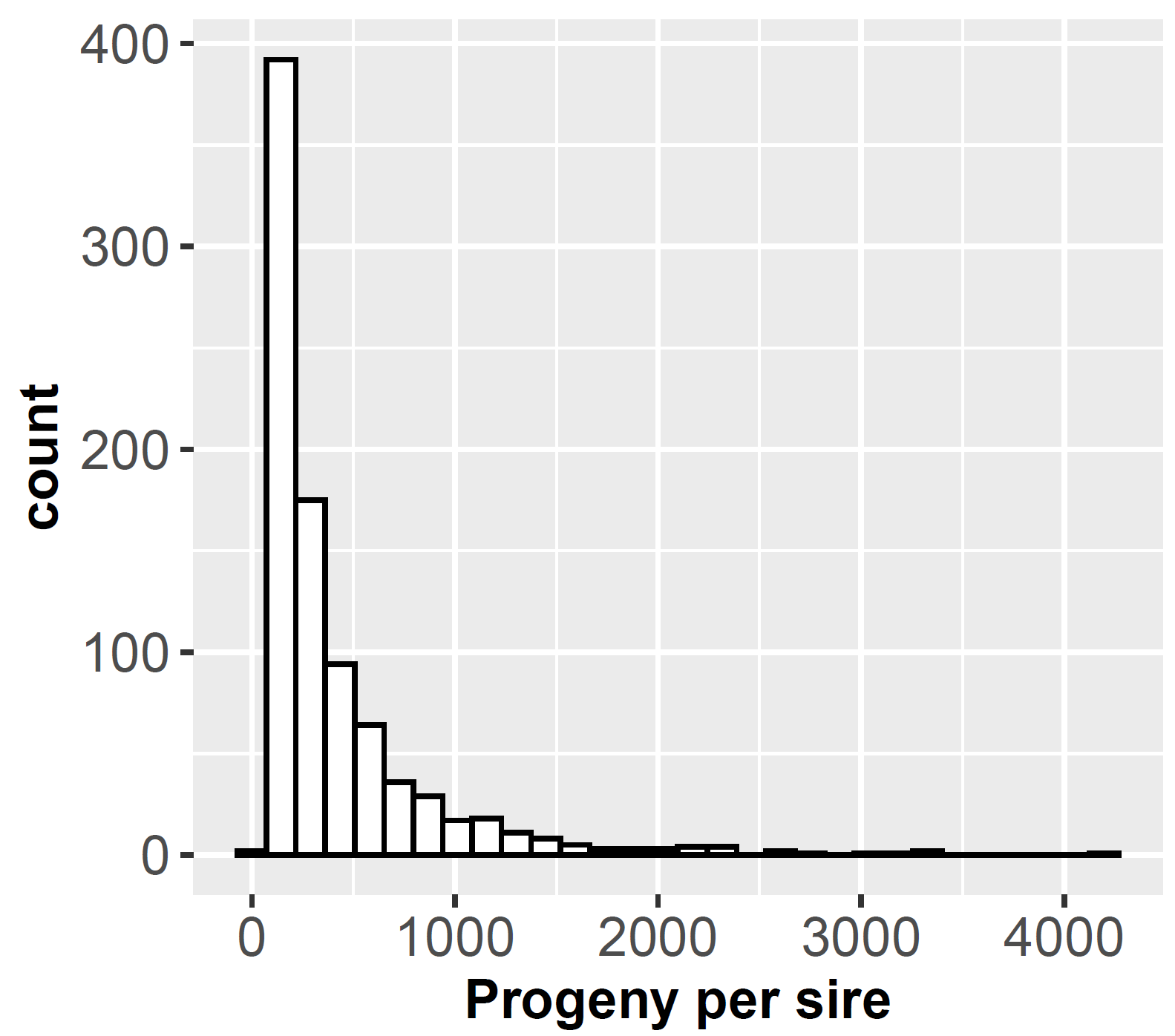

Supplement: Supplementary file 1 — Additional file 1: Figure S1. Number of progeny per sire of half-sib families. [file 12711_2020_593_MOESM1_ESM.tif]

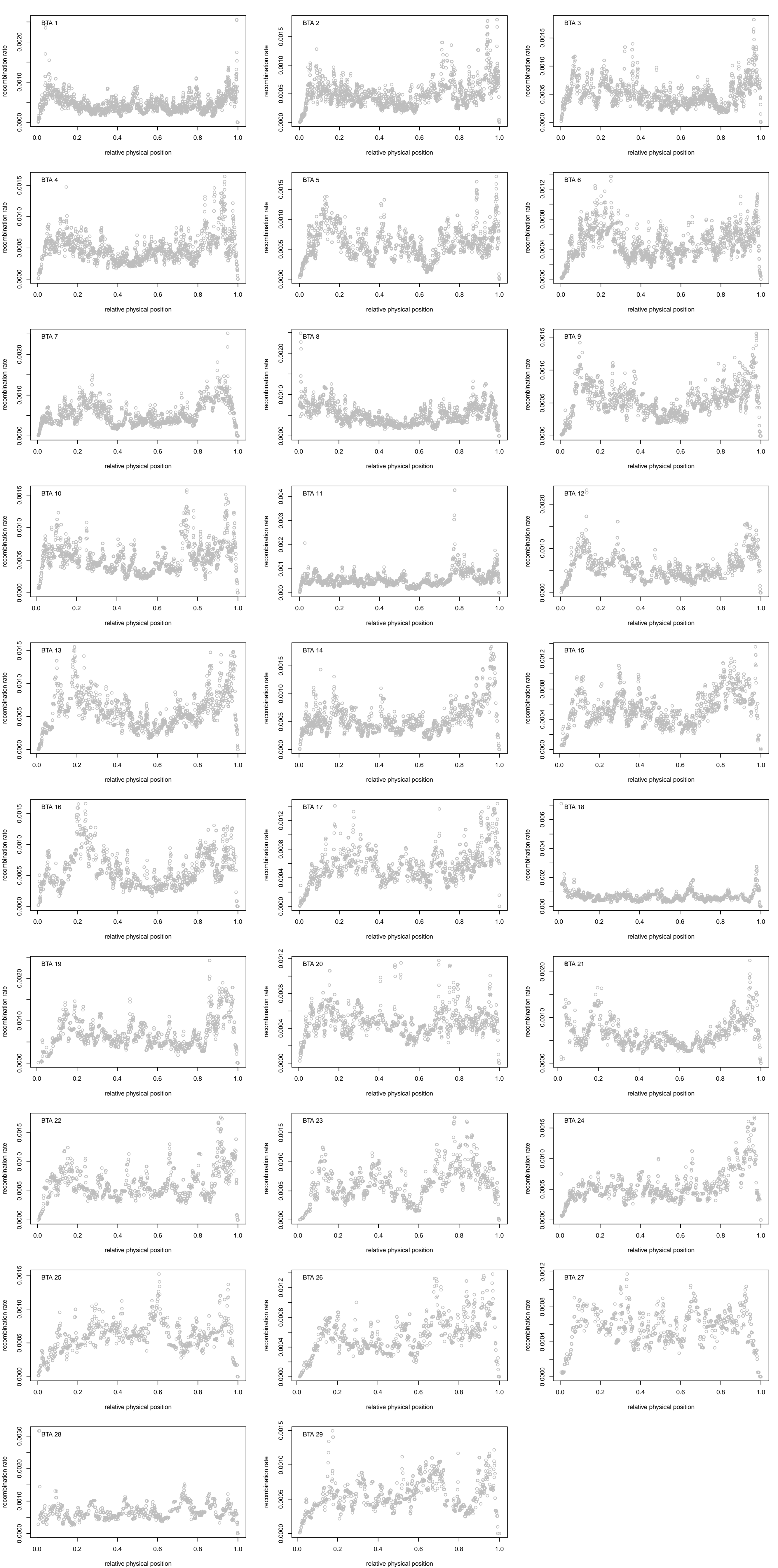

Supplement: Supplementary file 3 — Additional file 3: Figure S2. Illustration of the recombination rate along the chromosomes. The figure shows the relationship between recombination rate between adjacent markers based on the deterministic approach and the relative physical position for each chromosome. [file 12711_2020_593_MOESM3_ESM.pdf]

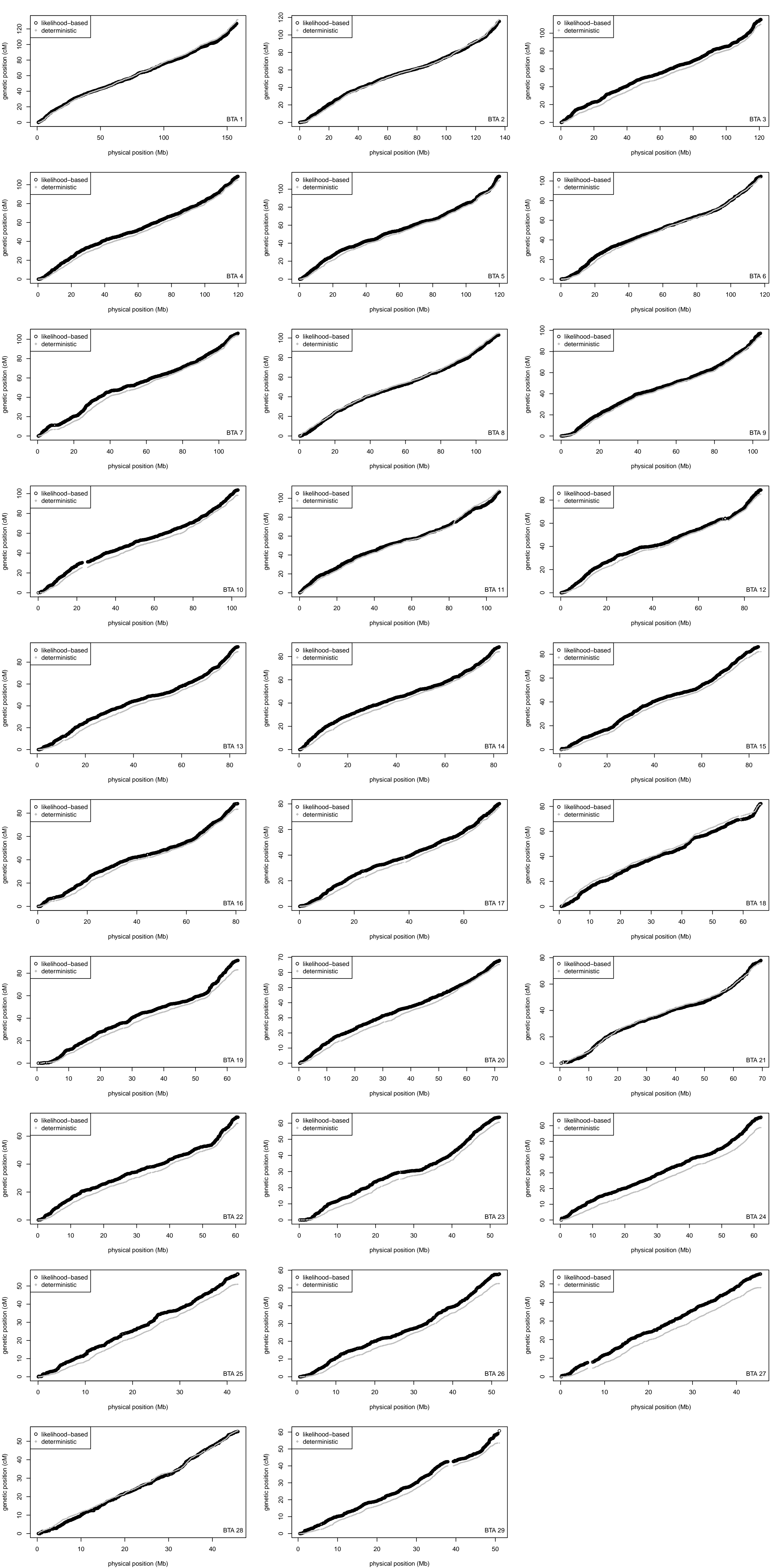

Supplement: Supplementary file 4 — Additional file 4: Figure S3. Physical genetic maps for each chromosome. The figure shows the relationship between physical and genetic-map coordinates for each chromosome. [file 12711_2020_593_MOESM4_ESM.pdf]
