# Supplementary material for: Male recombination map of the autosomal genome in German Holstein
Source: Genet Sel Evol. 2020 Dec 14;52:73. doi: 10.1186/s12711-020-00593-z (PMC7734841; doi:10.1186/s12711-020-00593-z)
Supplement: Supplementary file 6 — Additional file 6. Description of the simulation study to compare genetic map positions derived from the deterministic and likelihood-based approach [20–24, 35, 36]. [file 12711_2020_593_MOESM6_ESM.pdf]

SUPPLEMENTAL MATERIAL TO "MALE RECOMBINATION MAP OF THE AUTOSOMAL GENOME IN GERMAN HOLSTEIN"

by S. Qanbari and D. Wittenburg

Estimating genetic-map positions using simulated data

Likelihood-based approach

Exploiting the genetic similarity of paternal half-sibs, the recombination rate $\theta_{i,j}$ between markers *i* and *j* ($i, j = 1,\ldots,p;\ i < j$) can be assessed by using an established expectation-maximization approach (EM) which relies on likelihood theory [21-23]. Sire haplotypes and progeny genotypes are required. Then, having obtained all pairwise estimates $\hat{\theta}_{i,j}$ with the likelihood-based approach, we propose to approximate genetic length of each marker interval by a quadratic optimization approach employing all recombination rate estimates less than or equal to 0.05. For such small values, a linear relationship between recombination rate and genetic distance is assumed to hold. Let d_k denote the genetic distance between markers *k* and *k*+1. As genetic distances behave additive, e.g. $d_1 + d_2 + d_3$ is the genetic distance that corresponds to $\theta_{1,4} \leq 0.05$, we set up the optimization problem as follows

$$\min_{d_1,\ldots,d_{p-1}} \left\{ \sum_{\substack{i,j=1 \\ i<j \\ \hat{\theta}_{i,j} \leq 0.05}}^{p} \left(\hat{\theta}_{i,j} - \sum_{k=i}^{j-1} d_k \right)^2 \right\} \text{ s.t. } d_k \geq 0,\ k = 1,\ldots,p-1 \ .$$

The genetic length of a chromosome is calculated as the sum over interval lengths. This approach is implemented in the R package hsrecombi 0.3.0 as function geneticPosition [24] employing the function solve.QP from the R package quadprog 1.5-8 [35].

Data

To mimic an average bovine autosome, a chromosome covered by $p = 1{,}500$ biallelic markers on 100 Mbp length was simulated. This marker density is comparable to a 50K SNP chip. The first and last marker were placed at 0 and 100 Mbp, respectively. The remaining marker positions were drawn at random from a uniform distribution on [0, 100]. In total, 1,000 meioses were simulated. For each meiosis, the number of cross-over events (*k*) was drawn from a Poisson distribution following Haldane's theory [36] and assuming that chromosome length is approximately equal to $L = 1$ Morgan, i.e.

$$\Pr(x = k) = \frac{L^k e^{-L}}{k!} \ .$$

Afterwards, these *k* cross-overs were distributed uniformly over the chromosome.

A single paternal half-sib family was simulated. Sire genotypes at all markers were sampled at random considering allele frequencies drawn from a uniform distribution on [0.1, 0.9]. The 1,000 meioses built the paternal gametes of half siblings. Paternally inherited alleles were determined depending on the positions of cross-over events for each progeny following the scheme in Fig. S1. Maternally inherited marker alleles were drawn at random respecting the same allele frequencies. For convenience, maternal gametes were in linkage equilibrium. The simulation was repeated 10 times.

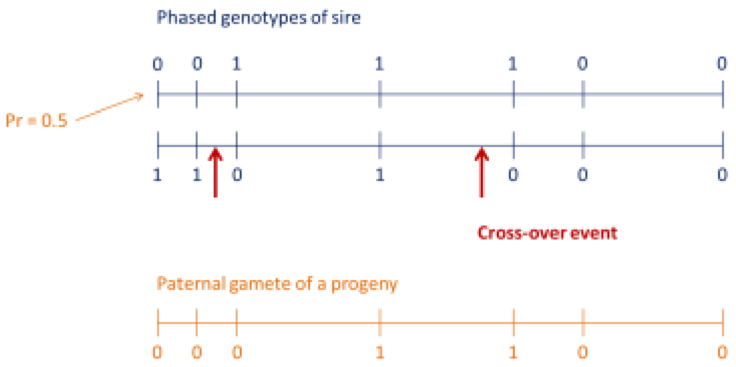

Figure S4. Construction of a paternal gamete.

In order to exclude uncertainty introduced by phasing the sire haplotypes, the simulated haplotypes were taken as input for the likelihood-based approach.

Evaluation

To obtain baseline values for evaluating the likelihood-based approach, cross-overs were also counted between marker pairs. An odd number of cross-overs between two markers constituted a recombination event. The relative number of recombinant offspring represented recombination rate.

Additionally, results were compared to a deterministic approach for the estimation of recombination rate between adjacent markers [20]. In this case, and because of the close proximity of markers, estimates were directly converted into genetic distances in Morgan units. The mean squared error of genetic distances served as a measure of precision.

Results

A linear relationship between physical and genetic distances has been ascertained which can be explained by the uniform distribution of cross-over events in the simulation study (selected examples in Fig. S2). The likelihood-based approach led to underestimated genetic distances but mean squared error was on average 19% less than compared to the deterministic approach. Chromosome length was estimated on average as 0.82 Morgan using the likelihood-based approach and as 0.76 Morgan based on hsphase. The number of estimates considered differed by one magnitude: on average 17,494 estimates of recombination rate have been included in the likelihood-based approach, whereas hsphase solely relied on 1,499 estimates between neighboring markers.

The average sire heterozygosity was 39%. The likelihood-based approach was almost unaffected by the degree of sire heterozygosity. In contrast, the performance of the deterministic improved only if sire heterozygosity was 100% which is an unlikely event (results not shown).

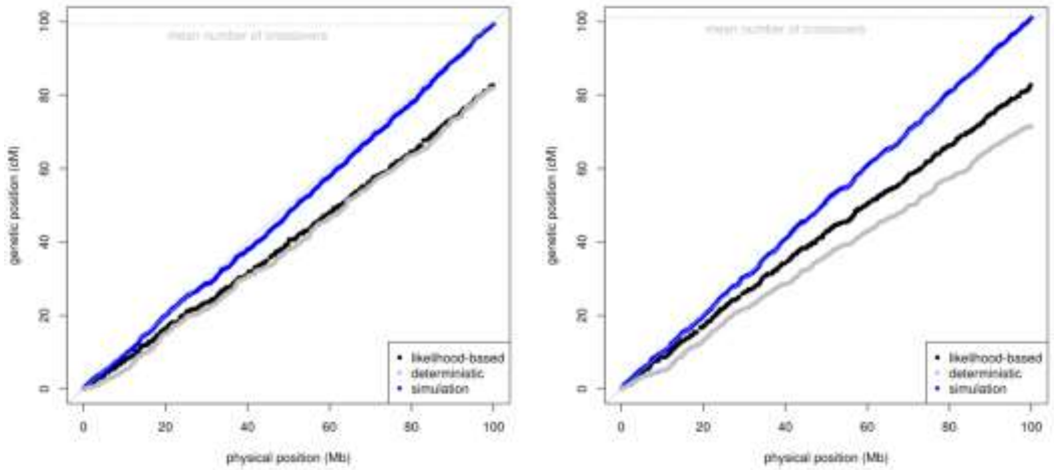

Figure S5. The physical-genetic map for a simulated chromosome in two repetitions. The dark blue line is based on counting recombinant offspring between adjacent markers. The black line was obtained by applying the optimization approach to likelihood-based estimates of recombination rate between any marker pairs. The grey line is the results of estimating recombination rate between adjacent markers with the deterministic approach hsphase. The dashed grey line marks the average number of simulated cross-over events in 1000 meioses.

Conclusion

The simulation study resembled a realistic set-up for the paternal contribution to genetic variation among half siblings. Genetic-map positions were determined from paternal recombination rates. Though the likelihood-based approach still led to underestimated genetic distances, the overall performance was superior to the deterministic approach of hsphase which exploited only a fraction of available information.

Transferring conclusions to real data investigation suggest that further improvements are necessary to cope with the consequent underestimation of genetic distances in general.
